# Circulating microRNAs in malaria infection: bench to bedside

**DOI:** 10.1186/s12936-017-1990-x

**Published:** 2017-08-15

**Authors:** Supat Chamnanchanunt, Suthat Fucharoen, Tsukuru Umemura

**Affiliations:** 1 Department of Clinical Tropical Medicine, Faculty of Tropical Medicine, Mahidol University, Bangkok, Thailand; 2 Thalassemia Research Center, Institute of Molecular Biosciences, Mahidol University, Bangkok, Thailand; 3 Department of Medical Technology and Sciences, International University of Health and Welfare, Ohkawa, Japan; 4 Department of Health Sciences, Graduate School of Medical Sciences, Kyushu University, Fukuoka, Japan

**Keywords:** Severe malaria, Plasmodium infection, MicroRNA (miRNA), Biomarker

## Abstract

Severe malaria has a poor prognosis with a morbidity rate of 80% in tropical areas. The early parasite detection is one of the effective means to prevent severe malaria of which specific treatment strategies are limited. Many clinical characteristics and laboratory testings have been used for the early diagnosis and prediction of severe disease. However, a few of these factors could be applied to clinical practice. MicroRNAs (miRNAs) were demonstrated as useful biomarkers in many diseases such as malignant diseases and cardiovascular diseases. Recently it was found that plasma miR-451 and miR-16 were downregulated in malaria infection at parasitic stages or with multi-organ failure involvement. MiR-125b, -27a, -23a, -150, 17–92 and -24 are deregulated in malaria patients with multiple organ failures. Here, the current findings of miRNAs were reviewed in relation to clinical severity of malaria infection and emphasized that miRNAs are potential biomarkers for severe malaria infection.

## Background

Malaria is a life-threatening arthropod-borne disease with high fatality rate in tropical countries. Almost one million patients with severe malaria are dying a year in the world [1]. The disease is caused by five distinct species of protozoa, namely *Plasmodium falciparum, Plasmodium malariae*, *Plasmodium ovale, Plasmodium vivax* or *Plasmodium knowlesi*. A majority of malaria infections are caused by *P. falciparum* (over 90%), followed by others species [2–5]. The 2000 World Health Organization (WHO) malaria case classification categorized the severity of malaria into severe (*P. falciparum)* and uncomplicated malaria [6, 7]. Diagnosis of severe malaria is evaluated if the condition is caused by *P. falciparum* infection with one or more of the followings: coma (cerebral malaria), metabolic acidosis, organ failure, and severe anaemia [8, 9]. High-risk factors for severe malaria are non-immune patients, immune-compromised patients, and those with a high burden of malaria parasites. A rapid increase of parasites in blood is the associated finding with severe malaria [10]. Thus, early parasite detection and the immediate start of treatment are key events to reduce severe form in malaria patients [11]. Microscopic examination of blood films, antigen detection, and molecular testing has been standard methods to detect malaria infection [7, 12]. Nowadays, microarray analysis and miRNA approach have helped many researchers to understand the relationship between dysregulation of miRNA and many infectious diseases [13–20].

MicroRNA assay need only a small amount of blood with less invasive sampling [21]. Furthermore, miRNAs have high sensitivity and specificity for the diagnosis of various disorders. Circulating miRNA carry the potential to predict the severe outcome and to improve patient care in malaria patients. This review describes circulating miRNAs as potential biomarkers for severe malaria infection.

## Type of miRNAs and limitations

The miRNAs testing started as the analysis of miRNAs obtained from cells or tissues. Recent studies have shown that many human body fluids contained miRNAs. Thus, body fluids are analysed as possible biomarkers to demonstrate the relationship between miRNAs and disease severity. There are still controversies about suitable sources to augment the usefulness of miRNAs as biomarkers [22, 23].

### Cellular miRNAs

**Fig. 1:**
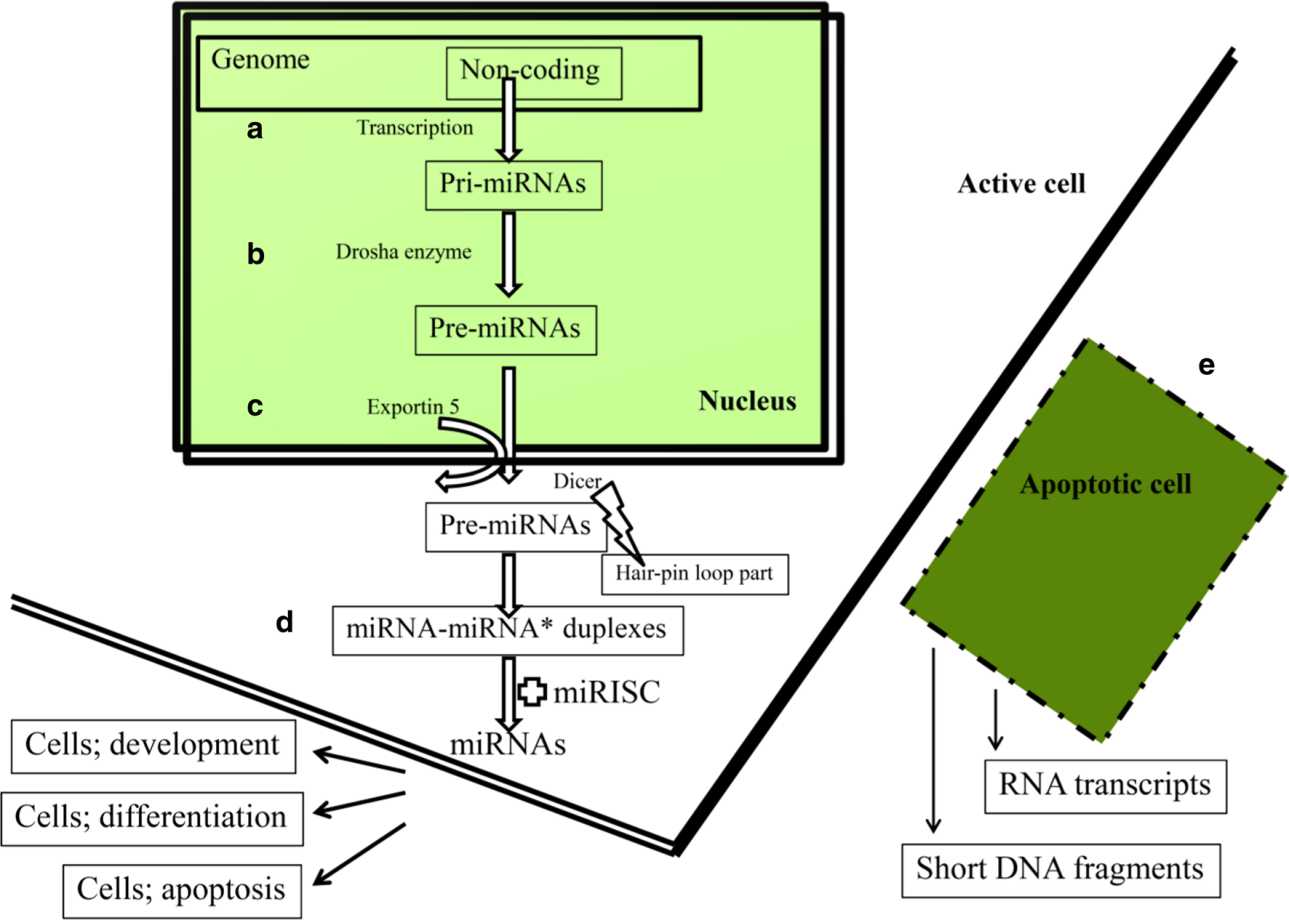
Biogenesis and apoptotic bodies of miRNAs. **a** The miRNA genes are located mainly in the non-coding region of the genome and are firstly transcribed to primary microRNAs (pri-miRNAs). **b** Drosha enzyme cut pri-miRNAs to precursor microRNAs (pre-miRNAs) (45–140 *nt*) [25]. **c** Pre-miRNAs are transferred out of the nucleus with Exportin 5 and are digested at the hair pin loop part of pre-miRNA with Dicer. **d** The miRNA–miRNA* duplexes are split into two asymmetric strands. Mature single strand miRNAs are finally bound to miRNA-induced silencing complex (miRISC) [29]. **e** MiRNAs suppress the expression of the target genes via mRNA cleavage or translation repression. The functions of miRNAs are involved in development, differentiation, and apoptosis of the cells [30]

MiRNA was firstly extracted from *Caenorhabditis elegans* tissues [24]. It is short nucleotides with 18–25 single-stranded RNA [25, 26]. The miRNA genes are located mainly in the non-coding region of the genome and are firstly transcribed to primary microRNAs (pri-miRNAs) [27, 28]. The miRNA was a synthesis in the nucleolus/cytoplasm and some released from apoptotic cells (Fig. 1) [25, 29, 30]. The early studies on miRNAs were done using organ or biopsy tissue. The limitation of testing may relate to tissue type, volume and extraction method. The discovery of miRNAs as promising disease biomarkers either in the blood or plasma was a breakthrough [31].

### Circulating miRNAs

Circulating miRNAs were studied in both plasma and serum. Mitchell et al. [32] found that good stability of miRNAs as the useful biomarker in both sample types. More recent studies have investigated their relationship between the pathophysiology of disease, in particular in malignancy or cardiovascular diseases [22, 33]. Mitchell et al. [32] also demonstrated that plasma contained miRNAs, and had functions to control gene expression. The correlation between circulating miRNAs and tissue miRNAs was found in this study. They also noted that the levels of circulating miRNAs were high enough to analyse using patient plasma.

Plasma miRNAs are circulating in four different molecular forms [34]. The first is microparticle (MP) miRNAs. Microparticles are vesicles with sizes of 100–1000 nm, produced from the cell surface by budding of the outer cell membrane. Both miRNAs and MP might act together to regulate immune status [35]. The second form is exosome miRNAs. Exosomes are released from multi-vesicular bodies and contain DNA, mRNA, proteins as well as miRNAs [34]. The size of exosome is 50–100 nm. The third form is miRNAs in apoptotic bodies of 1–5 μm produced in the apoptotic process of various cells. The fourth form is a protein or lipid-bound form including argonaute2 (Ago2) and high-density lipoprotein (HDL). All forms of miRNAs are stable in circulation, especially MP or exosome-included miRNAs [34, 36]. The levels of miRNAs in serum, saliva, and urine are lower than in plasma [37]. Therefore, saliva and urine sources of miRNAs need to use a large volume of sample to extracted miRNAs [36].

Kirschner et al. [38] described haemolysis of cells related to increasing of miRNAs level, which led to false negative findings to evaluate the levels of red blood cell-derived miRNAs. These effects were detectable in blood samples drawn from healthy individuals or in samples with delayed blood separation. Another factor to modify miRNA levels in plasma was co-extraction of cellular miRNAs from blood cells or cell debris. Membrane filters with low protein binding affinity were recommended, although the timing of filtration was still debated. In the case of unavailable tissue samples, it is a useful solution to detect miRNAs in blood or plasma samples. The analysis of circulating miRNAs is less invasive than tissue biopsy. Thus, these new molecules have been used in many diseases as the new biomarkers such as malignant diseases and cardiovascular diseases [39–41]. However, the knowledge about the usefulness of miRNA in infectious diseases is still not enough.

### MicroRNAs in infectious diseases

The evidence for interaction between host and pathogens via the miRNA pathway was documented in mammal infectious diseases [13]. Nukui et al. reported that miRNAs encoded by human herpesvirus 6A (HHV-6A) modulated the function of mammal cells. They demonstrated candidate miRNAs (miR-U86) regulating lytic replication of HHV-6A gene U86 [42]. Umbach et al. showed the latency associated transcript (LAT) was primary miRNA precursor to control four distinct miRNAs in herpes simplex virus. They reported miR-H2-3p related to viral HSV-1 replication by controlling viral gene expression at the latent period of infection [43]. Hook et al. found miR-UL112-1 related to viral target factors (UL114), which regulated cytomegalovirus to attach hematopoietic stem cells in bone marrow for reactivation and replication. Cytomegalovirus used miR-UL148D-1 and miR-UL112-1 to the target gene with the function of immune evasion. This action used RANTES, a chemokine to augment immune cells to infection site and prevented the attack from NK cells to infected cells [44].

For an infection that can lead to cancer, there were two studies identified the relationship between viral infection and malignancy. Motawi et al. and Bandiera et al. showed a similar result that hepatitis C virus (HCV) had miR-34 and miR-122 related with a chronic condition and more tendency to turn to hepatocellular carcinoma [14, 45]. Two candidate miRNAs can stimulate hepatocytic differentiation and cholesterol/fatty acid synthesis. Liver finally developed into fibrosis stage and malignancy at the high prevalence rate. The human miRNA profile is a useful tool for the diagnosis of organ dysfunction or infection, as well as malignancy. However, all studies were investigated for virus group in both pathophysiology and prognosis of diseases [13, 14, 42–45]. There were only limited studies on other infectious diseases, especially tropical diseases.

### Tropical diseases and miRNAs

The majority of miRNAs studies on tropical diseases were done in schistosomiasis, leishmaniasis, cryptosporidium, and toxoplasma infection. Firstly, He et al. and Zhu et al. identified miR-223 and miR-454 as important molecules, respectively, for pathogenesis in schistosomes infection [16, 46, 47]. The miR-223 had a function as the transcription regulator, transcription factor activity, DNA binding, and a role of miR-454 in progression via the TGF-β/Smad4 pathway in this parasite [16, 47]. In Leishmania infection, Geraci et al. demonstrated that the dysregulation of miR-21 and miR-146b-5p that were associated with *L. donovani*-infected monocyte-derived dendritic cells. Two miRNAs act via the tumor growth factor-beta (TGF-β) signaling pathway [18]. For protozoa infection, they found that decreased miR-221 levels were indicative of *Cryptosporidium parvum* infected epithelial cells with luciferase activity assay [19]. Saçar et al. [20] also reported that miR-328 levels relate to toxoplasmosis infection in human. The discovery of many parasite-specific miRNAs profiles can be used to apply in clinical practice. *Cryptosporidium* spp. and *Toxoplasma* spp. are Apicomplexa parasites, related to *Plasmodium* [48]. However, there has been a little body of knowledge of clinical applications of miRNAs in malaria infection.

## Malaria and miRNAs

### Identification of potential miRNAs and malaria infection

Malaria-infected RBCs can develop in malaria parasites compose with two asexual stages in human (blood steam and liver) and sexual stage in the mosquito. In human, these parasites invade into human red blood cells (RBCs) until the mature parasite sequestration using cytoadherence ligand of *P. falciparum* Erythrocyte Membrane Protein-1 (PfEMP-1) [49, 50]. Splenic macrophages are the main of clearance of malaria from blood circulation. The miRNA possibly helps malaria to invade and grow in RBCs via escape from immune responses and defect of opsonization by circulating macrophage [49, 51–53]. Analysis of plasmodium genome demonstrated more than 500 genes [54]. Two groups reported that *P. falciparum* did not have miRNA-sequences in parasite genome [50, 55]. They made a clone of all RNAs from a mixed stage of malaria-infected RBCs and then tested with bioinformatics method. It showed no matching between those cloned sequences and miRNA structures. There has been no study showing the presence of RNAi-family [siRNA, miRNAs, repeat-associated small interfering RNAs (rasiRNAs), and PIWI-interacting RNAs (piRNAs)] according to the stage of the parasite. In some infectious diseases, interactions between host miRNA and pathogen gene, or vice versa, were reported. For examples, human miRNAs including miR-223 suppress human immunodeficiency virus-1 (HIV-1) mRNA [56], Epstein-Bar virus miRNA: miR-BHRF1-3 targets human interferon (IFN)-inducible T cell-attracting chemokine (CXCL11) gene expression [57], and human miR-122 targets to hepatitis C virus (HCV) RNA [58]. However, it is still not known whether human miRNA interacts with malaria mRNAs. Further examination may bring the miRNA-based diagnosis or therapeutic approaches. The posttranscriptional gene silencing in *Plasmodium* parasites changes alternative pathways other than miRNAs. The possibility is that *Plasmodium* is utilizing host miRNAs to regulate their gene expression [49, 59, 60].

The correlation between reported miRNAs was reviewed according to malaria parasite species (Table 1). For *P. falciparum* malaria, Rathjen et al. found that the block of miR-451 synthesis pathway by knocking out Ago2 which produces mature miR-451 resulted in the development of severe anaemia in mice [55]. Similar to the previous study that miR-451 is an essential molecule for erythroid cells since miR-451 was up-regulated during human erythroid differentiation [61]. Xue et al. [50] also showed that 36 clones of miRNA were found in infected erythroid cells, not in malaria parasite and the majority of genome composes with 80–90% of A-T rich sequence in *P. falciparum* parasites. Both studies did not found any *Plasmodium* specific miRNAs; those might be an effect from cells culture method. In erythroid cells, LaMonte et al. and Chapman et al. found that the levels of miR-233 and miR-451 were high in these parasite-infected cells when compared with normal [49, 62]. They suggested that impaired growth of parasites might be resulted from a block of mRNA translation by miR-451 and miR-223 in human red blood cells. Thus, Rathjen et al. and Xue et al. demonstrated that parasite could diminish miR-451 level in serum, but be accumulated in *Plasmodium*-infected RBCs. Similar to Chamnanchanunt et al. observed the lower levels of miR-451 and -16 in serums from 22 *P. vivax* patients than non-infected subjects [63]. This group also found downregulation of miR-451 and -16 in red blood cells of *P. vivax* patients [64]. Reducing miR-451 relates to Ago2 in extracellular vesicles (EVs) to stimulate oxidative damage in infected-RBCs [65]. Interestingly, Baro et al. [66] demonstrated that miR-221/222, -24 and -191 were decreased in bone marrow in *P. vivax* malaria patients. The numbers of *P. falciparum* patients need to more large scale study.

**Table 1:** Summary of discovery miRNAs among patients and animal experimental studies

Author/(reference)	Year	Study population	Down regulation	Up regulation
Human specimen				
Rathjen et al. [55]	2006	*P.f.* parasite in cell culture	miR-451: significantly accumulated in infected RBCs	
Xue et al. [50]	2008	*P.f.* infected in human erythroid cells	miR-451, let-7b, miR-16, miR-91, miR-142, miR-144, let-7a, let-7f, miR-92, miR106: identified form infected RBCs	
LaMonte et al. [49]	2012	HbAS and HbCC RBCs with *p. f*.	–	miR-451 and miR-223
Chamnanchanunt et al. [63]	2015	Patients with *p.f*. and *p.v.* infection	miR-451 and miR-16 (plasma of *p.v*. patients than *p.f*. patients)	–
Chamnanchanunt et al. [64]	2015	Patients with malaria infection	miR-451 and miR-16 (RBCs of *p.v*. patients)	–
Animal specimen				
Delic et al. [68]	2011	*P. chabaudi* infected in mice model	miR-10b, let-7a, let-7 g, miR-193a-3p, miR-192, miR-14205p, miR-465d, miR-677, miR-98, miR-694, miR-142-5p, miR-465d, miR-677, miR-98, miR-694, miR-374, miR-450b-5p, miR-464, miR-377, miR-20a, miR-466d-3p: (in liver)	miR-26b, miR-M23-1-5p, miR-1274a: (in liver organ)
El-Assaad et al. [69]	2011	*P. berghei* infected in mice model	–	let-7i, miR-27a, miR-150 (in brain organ)
Al-Quraishy et al. [67]	2012	*P. chabaudi* infected in mice model	miR-194, miR-192, miR-193A-3P, miR-145, miR-16, miR-99A, miR-99B, miR-15A, miR-152, let-7G, let-7B, miR-455-3P: (in spleen and liver)	–

For an animal model of demonstration parasite induces organ failure, mice infected with *P. chabaudi* malaria showed that 12 common miRNAs were downregulated in spleen and liver tissues [67]. A study by Delic et al. found three miRNA species upregulated and 16 miRNA species downregulated [68]. These findings suggested that miRNAs might be reprogrammed to minimize disease severity after infection. Knowledge of the interaction between falciparum parasite and the human genome could be valuable in malaria control. Furthermore, a study by El-Assaad et al. found that mouse with cerebral malaria had overexpression of miR-27a, miR-150, and let7i levels in brain tissue compared to a mouse with no cerebral malaria [69]. Thus, miRNAs would have significant roles as biomarkers to predict early host responses and prognosis of malaria infection.

The knowledge of miRNAs as possible disease biomarkers in blood is a promising breakthrough especially the patients with malaria infection. For practical use, the disease criteria for severe falciparum malaria were applied to identify severe falciparum malaria patients from non-severe form [70] (Table 2). The relationship between candidate miRNAs and severe falciparum malaria is not yet clearly understood, and this might help to predict early critical patients.

**Table 2:** Criteria for severe or complicated falciparum malaria infection [4–7] and candidate miRNAs

Categories	Clinical or laboratory to diagnosis condition	Postulated miRNAs	Mechanism
Acidosis/acidemia	Artrial pH <7.3 or presence of acidosis	miR-210	HIF-dependent trasncriptional regulation
ARDS or pulmonary edema	The acute lung injury from noncardiogenic causes	miR-181b	NF-kB mediated vascular inflammation
		miR-125b	LPS-induced lung injury
Cerebral malaria	Imparied consciousness or seizures	miR-210	Regulation of the revascularization
		miR-27a, miR-23a	Brain activation by EFNA3, NP1
		miR-150	Stimulate angiogenic factors
Renal failure	Urine output <0.4 ml/kg/hour or serum creatinine >3.0 mg/dl	miR 17–92	Renal progenitors and renaly dysfuntion
		miR-24	Apoptosis regulation
Ongoing investigation			
Anemia	Haemoglobin ≤8 g/dl	n.a.	–
Shock	Blood pressure <90/60 mmHg with the sign of cold, clammy extremities	n.a.	–
DIC	The presence of DIC phenomenon or spontaneous mucosal bleeding	n.a.	–
Hyperparasitemia	Presence of parasitized erythrocytes >10%	n.a.	–
Hypoglycemia	Presence of blood sugar <40 mg/dl	n.a.	–
Macroscopic hemoglobinuria	The presence of hemolysis in the patients without G6PD deficiency	n.a.	–

*ARDS* Acute respiratory distress syndrome, *DIC* Disseminated intravascular coagulopathy, *G6PD* glucose-6-phosphate deficiency, *n.a.* not available data, *HIF* hypoxia-inducible factor, *LPS* lipopolysaccharide, *EFNA3* epihrin-A3, *NP1* neuronal pentraxin

## Candidate miRNAs in severe malaria with multi-organ failure

### Acidosis severe malaria

Patients with severe malaria infection develop acidosis as a serious complication [71]. It comprised with various mechanisms directly target the redox status and tissue hypoxia. Grosso et al. and Ivan et al. showed that miR-210 led to augmented hypoxia-inducible factor (HIF)-dependent transcriptional regulation and hypoxic condition [72, 73]. Further study needs to underline the mechanism of miR-210 in the area of malaria infection.

### Pulmonary complications

Acute respiratory distress syndrome (ARDS) is the major complication of severe malaria [6, 74]. ARDS is the defect of gas exchange on the lung and pulmonary/alveolar capillary permeability [6, 75]. Sun et al. found that low levels of miR-181b in patients with ARDS, which regulates NF-kB, mediated vascular inflammation of the lung [76]. Guo et al. observed that miR-125b was downregulated in ARDS patients. MiR-125b was reported to be related LPS-induced lung injury [77].

### Neurological complications

Cerebral malaria is the condition of alternation of consciousness due to parasite sequestration and brain hypoxia [78]. The hypoxic condition occurs as the results from apoptosis, microvascular occlusion, cytoadhesion from parasitized RBCs aggregates in brain vessels, and then lead to high lactate levels in cerebrospinal fluid, hypoperfusion or constriction of small vessels [69]. Lou et al. reported the upregulated levels of miR-210 (involved in the regulation of the revascularization) in hypoxic condition. Sabirzhanov et al. showed downregulated miR-23a and miR-27a that injured cortex after traumatic brain injury [79, 80]. He et al. [17] demonstrated that high levels of miR-150 in the brain during cerebral ischaemia that could directly regulate the angiogenic factors. Moreover, a hypoxic condition induced upregulation of miR-210 in the patients with malignancy and cerebrovascular diseases [81]. The explanation of miR-210 in hypoxia-induced acidosis related to the action of Ephrin-A3 (EFNA3), and neuronal pentraxin 1 (NP1) in the brain. Another study by Krupinski found that miR-120 levels have a positive correlation between an encephalopathy with lactic acidosis patients [82]. These reports showed that miRNAs are relevant biomarkers for brain damage. There are no observations yet on miRNAs involved in brain malaria patients.

### Renal complications

Kidney failure is a common complication in severe malaria patients [6]. The mechanism to develop renal damages composes with multiple factors (cytoadherence from infected RBCs leads to obstruction, hypovolaemia from body fluid loss and host immune responsiveness) [83]. Marrone et al. found that down-regulation of miR-17–92 related to renal progenitors and renal dysfunction in adult mice with acute nephropathy [84]. Lorenzne et al. [85] demonstrated that high levels of miR-24 in the ischemic kidney mice. The effect on apoptosis regulation is an explanation for this miRNAs action. However, the significant miRNAs relate to renal dysfunction after malaria infection is not defined.

## Summary and perspectives

The optical microscopic examination is still the gold standard to detect malaria parasites. This method is straightforward and cheap. There are no currently available diagnostic techniques to predict the malaria severity that is very important for saving patient’s life. The new technology has more potential to help the physicians to manage severe malaria is recommended. New investigate applied into tropical diseases such as malaria infection. In vivo study of malaria infection was turn into clinical application. In addition, as a biomarker, it would have prognostic value, especially in early host responses. The objective of this review literature is to systematically review the available data on the relevance of candidate miRNAs among both in vitro and in vivo malaria study.

A useful biomarker has to be an investigation, with high sensitivity, non-invasive and applicable to clinical management. There are more studies about miRNAs as a biomarker in many diseases, most of them related to malignant and cardiovascular diseases. For tropical infectious diseases, the knowledge of miRNAs raises to fill to explain the pathophysiology of diseases. The knowledge of miRNAs in malaria infection is still not enough if compared with antimalaria drug trial. The more studies were required to answer, (1) biomarker to predict malaria severity and further predictor, (2) development of the new treatment directed to malaria life cycle. Further studies will perform to fill the gap between bench and bed by accumulating knowledge in serious complications among malaria patients.
